# Effectiveness and safety of Chinese traditional medicine Ulcer Ointment for skin ulcers: a systematic review and meta-analysis of randomized controlled trials

**DOI:** 10.3389/fphar.2026.1764562

**Published:** 2026-03-12

**Authors:** Bingrui Zhang, Wenying Wang, Shengxian Wu, Baochen Zhu, Lei Chen, Fengtong Liu, Xiaoran Li, Dongyang Lin, Mingyue Liu, Xi Li

**Affiliations:** 1 Dongzhimen Hospital, Beijing University of Chinese Medicine, Beijing, China; 2 Beijing Friendship Hospital, Capital Medical University, Beijing, China

**Keywords:** diabetic foot ulcers, meta-analysis, skin ulcers, systematic review, traditional Chinese medicine, venous leg ulcers

## Abstract

**Introduction:**

Ulcer Ointment (UO), a topical agent derived from traditional Chinese medicine, has been widely used for skin ulcers. This review evaluates its effectiveness and safety.

**Methods:**

We systematically searched eight databases for randomized controlled trials (RCTs) focusing on UO for skin ulcers. Pooled mean difference (MD) and relative risks (RR) with 95% confidence intervals (CI) were calculated.

**Results:**

Fourteen RCTs involving 978 participants with diabetic foot ulcers, venous leg ulcers, acutely infected ulcers, and pressure ulcers were included. Overall study quality was low. Compared with no intervention, UO was associated with a higher healing rate (RR = 2.24, 95% CI: 1.42–3.52, 2 RCTs, n = 140), reduced ulcer area, shorter healing time, lower pain scores, and elevated vascular endothelial growth factor (VEGF) levels. UO were more efficacious than standard topical drugs in healing rate (RR = 1.87, 95% CI: 1.49–2.34, 8 RCTs, n = 462), percentage reduction in ulcer area (RR = 17.82%, 95% CI: 12.63–23.00, 3 RCTs, n = 179), ulcer area (RR = −1.66 cm^2^, 95% CI: −1.98 to −1.35, 3 RCTs, n = 157), healing time, clinical effective rate (RR = 1.21, 95% CI: 1.10–1.32, 9 RCTs, n = 491), TCM symptom complex scores, pain scores, and VEGF levels. Although these differences are statistically significant, the clinical reliability of these benefits remain uncertain. No severe adverse events were reported in the UO group.

**Conclusion:**

Based on the currently available low-quality evidence, UO has shown preliminary indications of potential benefits in ulcer healing, improvement of TCM symptoms, pain alleviation, and elevation of VEGF levels. However, the exact efficacy of UO for skin ulcers requires further validation through high-quality double-blind RCTs.

## 1 Introduction

Skin ulcers are characterized by a full-thickness loss of the epidermis and dermis, often extending into deeper tissues such as the subcutaneous fat, muscle, or bone. They result from a persistent interruption in the normal wound-healing process, leading to a chronic, non-healing wound (Mustoe et al., 2006). Based on the primary underlying etiology, skin ulcers are systematically classified into several categories: venous ulcers, ischemic ulcers, diabetic ulcers, pressure ulcers. According to a recent Europe-wide survey, over one-third wounds were categorized hard-to-heal (Milne et al., 2020). Skin ulcers impact the quality of life of about 2.5% of the United States population, and the management of ulcers has a significant economic impact on healthcare (Sen, 2021). Some types of ulcers, such as diabetic foot ulcers, have a 5 years mortality and direct costs similar to that of cancer (Armstrong et al., 2020). Skin ulcers constitute a growing burden on healthcare systems and patient wellbeing, a trend expected to accelerate with demographic shifts toward an older, multimorbid population (Huai et al., 2025).

Multifactorial pathologic states result in the development of skin ulcers, including arterial or venous insufficiency, diabetes, persistent skin pressure, presence of a foreign matter, and infection (Jones et al., 2018). Tissue hypoxia, persistent infection, and dysregulated inflammation lead to persistence of ulcers (Martin and Nunan, 2015). The healing of skin ulcers is a dynamic, multi-phase process encompassing hemostasis, inflammation, proliferation, and tissue remodeling, in coordinated efforts of keratinocytes, fibroblasts, vascular endothelial cells, and immune cells (Martin and Nunan, 2015). Conventional strategies for promoting skin ulcer healing include debridement, wound dressings, infection control, negative pressure wound therapy, lower extremity arterial revascularization, and skin grafting (Armstrong et al., 2023). Beyond these, emerging treatment methods such as nanotherapeutics, stem cell therapy, 3D-bioprinted skin, and extracellular matrix-based scaffolds offer innovative solutions (Kolimi et al., 2022).

For decades, botanical drugs have been valued for treating skin ulcers by leveraging the anti-inflammatory, antimicrobial, and cell-stimulating activities in a concerted action to promote healing (Pereira and Bártolo, 2016). Various traditional Chinese medicines have been used to treat skin ulcers since ancient times (Ning et al., 2022). The earliest traditional Chinese medicine (TCM) classic dedicated to this condition is *Liu Juanzi Guiyi Fang* from the Southern Qi Dynasty (479–502 AD), which documents formulas for skin ulcers of both internal and external use. Guided by TCM theory, clinicians individualized prescriptions based on pattern differentiation and clinical symptoms (Li et al., 2012). Among the many treatments developed through this practice, some fixed formulas have gained prominence for their recognized efficacy. The Chinese traditional medicine Ulcer Ointment (UO) is a topical agent originally developed at Dongzhimen Hospital, Beijing University of Chinese Medicine (Zheng et al., 2014). Its origins can be traced to the ancestral UO formula of the renowned TCM practitioner, Professor Shi Hanzhang. Having been applied in clinical practice for over 50 years, UO was officially standardized into a hospital-prepared proprietary Chinese medicine by Dongzhimen Hospital in 2005, utilizing modern decoction and sterilization techniques. The formulation comprises *Rheum palmatum* L. [Polygonaceae; *Rhei radix et rhizoma*], *Angelica dahurica* (Fisch. ex Hoffm.) Benth. & Hook.f. ex Franch. & Sav. [Apiaceae; *Angelicae dahuricae radix*], *Ligusticum chuanxiong* Hort. [Apiaceae; *Chuanxiong rhizoma*]. With primary functions of clearing heat and removing toxins, circulating blood and regenerating new tissues, as well as unblocking collaterals and moisturizing the skin, it has long been used to effectively manage various skin ulcers, including those that are chronic, non-healing, or acutely infected, as well as skin dryness and fissures.

Despite reports of efficacy in some trials, the absence of a systematic review prompted us to conduct a meta-analysis and evaluate the evidence base for UO. This study evaluated the effectiveness and safety of UO through outcomes such as healing rate, ulcer size, and pain scores, while also assessing the risk of bias and providing methodological recommendations for future research.

## 2 Methods

### 2.1 Registration

The protocol of this review was registered via PROSPERO (CRD420251177748) on 25 October 2025 (Available from: http://www.crd.york.ac.uk/PROSPERO/). This systematic review followed the PRISMA 2020 guideline (Supplementary Table S1).

### 2.2 Criteria for inclusion and exclusion

#### 2.2.1 Type of studies

This systematic review included randomized controlled trials (RCTs) comparing UO with a control (e.g., placebo, biomedical topical agents, or no intervention).

#### 2.2.2 Type of participants

Patients diagnosed with specific skin ulcers, based on the current comprehensive criteria or definitions of the International Classification of Diseases (ICD-11), were included. These include: (a) Diabetic foot ulcer (Armstrong et al., 2023): Patients with newly diagnosed diabetes or a history of diabetes present with foot ulcers, often accompanied by diabetic peripheral neuropathy and/or peripheral arterial disease. (b) Venous leg ulcer (Wittens et al., 2015): chronic skin ulcers of ankle or lower leg due to chronic peripheral venous hypertension. (c) Ischemic ulcer (Ouriel, 2001): Skin ulcer attributed to peripheral arterial disease. (d) Pressure ulcer (Mervis and Phillips, 2019): Ulcers resulted from localized injury and ischemic necrosis of skin and underlying tissues due to prolonged pressure. (e) Traumatic ulcer (Jagdeo and Shumaker, 2017): Ulcers attributed to external trauma. Patients at any phases of the disease are included. There are no restrictions on age, gender, or ethnicity.

#### 2.2.3 Type of intervention

The experimental intervention consisted of UO topically applied to the ulcer site, either alone or in conjunction with standard treatments such as debridement, systemic antibiotics, vasodilators, and analgesics. The UO was prepared by frying a 1:1:1 mixture of *Rheum palmatum* L. [Polygonaceae; *Rhei radix et rhizoma*], *Angelica dahurica* (Fisch. ex Hoffm.) Benth. & Hook.f. ex Franch. & Sav. [Apiaceae; *Angelicae dahuricae radix*], and *Ligusticum chuanxiong* Hort. [Apiaceae; *Chuanxiong rhizoma*] in sesame oil until brittle. The resultant mixture was then filtered and sterilized. Included studies must report the composition of UO in detail, using only botanical drugs that are monographed in a recognized national or regional pharmacopoeia. Control interventions included standard topical drugs (e.g., ethacridine lactate, recombinant human epidermal growth factor (rhEGF); excluding other TCM topical agents), placebo, or no intervention (e.g., normal saline, sterile dressing). Comparisons were made between the UO and control interventions. Both the UO and control interventions could be administered either as standalone treatments or alongside the same co-interventions (such as debridement, systemic antibiotics, vasodilators, or analgesics), provided that any additional treatments were identical between the experimental and control groups.

#### 2.2.4 Type of outcomes

A core outcome set (Staniszewska et al., 2024) developed using the COMET methodology recommends healing rate, healing time, health-related quality of life, and mortality for assessing interventions in skin ulcers. Building on this and considering that pain and other symptoms significantly impact patient quality of life (Ghadeer et al., 2025), we selected the following outcomes: healing rate, healing time, ulcer area, pain scores, and general symptom scores. The percent change in ulcer area is a validated, robust predictor of ulcer healing, establishing it as a pivotal measure of treatment response (Sheehan et al., 2006). The clinical effective rate, which was frequently used in skin ulcer trials, was defined for this study as a reduction in ulcer size accompanied by an alleviation of symptoms. Vascular endothelial growth factor (VEGF) can accelerate ulcer healing by stimulating angiogenesis, which restores blood flow, delivers oxygen and nutrients, and facilitates the formation of granulation tissue at the ulcer site (Singer and Clark, 1999).

Primary outcomes included the following: (1) Healing rate; (2) Percent change in ulcer area. Secondary outcomes included the following: (1) Ulcer area; (2) Healing time; (3) Pain scores (Williamson and Hoggart, 2005) (e.g., Verbal Analogue Scale, Numerical Rating Scale); (4) General symptom scores (e.g., TCM symptom complex score (Xiao et al., 2024), Quality of Life Scale); (5) Clinical effective rate; (6) Serum VEGF levels; (7) Adverse events.

### 2.3 Search strategy

We searched five Chinese databases (China National Knowledge Infrastructure (CNKI), Wanfang Database, Chinese Scientific Journal Database (VIP), SinoMed, and Yiigle Database) and three English databases (PubMed, EMBASE, and The Cochrane Library) from their inception to 28 October 2025, for relevant journal articles, conference papers, and academic dissertations published in Chinese or English. The main search terms included “ulcer,” “Rheum palmatum L.,” “Angelica dahurica,” “Chuanxiong,” “random*.” The specific search strategies for each database are detailed in Supplementary Table S2. We hand-searched relevant studies for additional eligible RCTs.

### 2.4 Study selection and data extraction

First, two reviewers independently screened the titles and abstracts using EndNote 20 software to identify potentially eligible studies, the full texts of which were then retrieved for further assessment. Subsequently, two reviewers independently extracted data from the included studies using a pre-designed data extraction form (including publication year, funding, inclusion and exclusion criteria, diagnostic criteria, participant characteristics, details of the interventions and controls, and outcomes). Any disagreements were resolved through consensus or by consulting a third senior reviewer.

### 2.5 Risk of bias assessment

The risk of bias was assessed independently and in duplicate using the Cochrane Risk of Bias Tool 2.0 (Sterne et al., 2019), which evaluates five domains: randomization process, deviations from the intended interventions, missing outcome data, measurement of the outcome, and selection of the reported result. Each domain was rated as “low,” “some concerns,” or “high.” The overall risk of bias for each trial was determined by its highest-risk domain. Studies that merely stated “randomized” without providing methodological details for the randomization process were judged as having “some concerns” in that domain. Any discrepancies in assessments were resolved through consensus or by consulting a third senior reviewer.

### 2.6 Data analysis

We conducted meta-analyses using RevMan 5.4.1 software. For continuous outcomes, we calculated the mean difference (MD) with a 95% confidence interval (CI) when the same measurement tool was used across studies, and the standardized mean difference (SMD) with a 95% CI when different measurement tools were employed. Dichotomous outcomes were expressed as relative risk (RR) with a 95% CI. Data that could not be pooled in a meta-analysis were summarized descriptively. Heterogeneity was quantified using the I^2^ statistic. Due to anticipated clinical heterogeneity (e.g., differences in UO’s production batch, treatment frequency, and duration), a random-effects model was applied for all data syntheses. If sufficient data were available, we planned to perform subgroup analyses based on: (1) ulcer duration (within 1 month, over 1 month); (2) types of skin ulcers (e.g., diabetic foot, venous leg, ischemic); (3) TCM patterns; and (4) treatment duration (within 1 month, over 1 month). Sensitivity analyses were planned to explore sources of substantial heterogeneity (I^2^ > 50%), based on study quality and key intervention characteristics. Publication bias was assessed using funnel plots and Egger’s test for outcomes that included ≥10 studies. The certainty of the evidence for each outcome was evaluated using the GRADE approach (Guyatt et al., 2013), which classifies evidence as high, moderate, low, or very low.

## 3 Results

### 3.1 Study identification and characteristics

**FIGURE 1:**
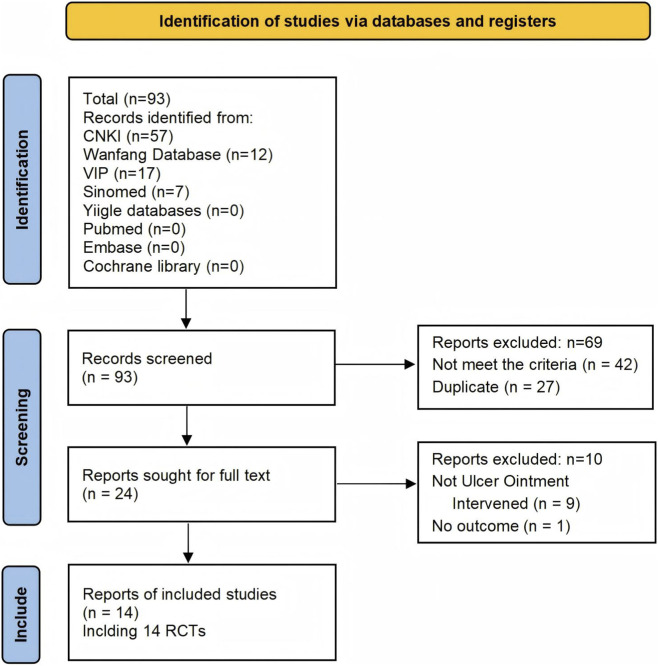
Prisma flow diagram of study selection process. CNKI, China National Knowledge Infrastructure; EMBASE, Excerpta Medica database; TCM, traditional Chinese medicine; VIP, Chinese scientific journal database; RCT, randomized controlled trial.

We identified 95 articles and finally included 14 RCTs (Jia, 2016; Lin et al., 2024; Lin, 2015; Lou, 2023; Wang, 2008; Wang et al., 2016; Wang et al., 2024; Xiao, 2016; Yang A.H., 2015; Yang X.X., 2015; Yi, 2015; Zhang, 2013; Zhao, 2012; Zhu, 2014) involving 978 adult patients with skin ulcers; the screening process is detailed in Figure 1. The included studies were all published in Chinese, and eight trials were conducted at Dongzhimen Hospital, Beijing University of Chinese Medicine, China. Table 1 presents the details of the included trials. In total, the trials enrolled patients with diabetic foot ulcers (8 RCTs), venous leg ulcers (4 RCTs), acutely infected ulcers with a duration of 1–3 days (1 RCT), and either diabetic foot or pressure ulcers (1 RCT). Three trials (Wang, 2008; Yang A.H., 2015; Yi, 2015) included patients with a damp-heat TCM pattern, while the remaining trials had no TCM pattern-related inclusion criteria. The sample sizes ranged from 29 to 160. The male-to-female ratio was 544:353, and two trials failed to report the participants’ gender. The overall age range is broad (18–90 years), but the majority of participants were middle-aged and older, with mean ages typically between 50 and 70 years. Very few studies included adults under 40, and even in those studies the average age remained near or above 40. Baseline ulcer size was reported in five trials, ranging from approximately 1–18 cm^2^. All the UO were hospital-prepared with the same composition and similar preparation process. Detailed information on the crude drug processing, extraction methods, production approval numbers, and manufacturers of each UO preparation is provided in Supplementary Table S3. They were applied topically to the ulcer site, covered with sterile gauze, once daily or every other day. Most trials performed routine debridement and disinfection prior to UO application. A total of 11 trials compared UO with standard topical drugs (e.g., ethacridine lactate, rhEGF, metronidazole, or petrolatum gauze). Only three trials directly compared UO with a control of normal saline or sterile dressing, which was defined as no intervention (Lin et al., 2024; Lin, 2015; Xiao, 2016). The total dropout rate was low, occurring in only three trial (Wang et al., 2024; Yang A.H., 2015; Zhao, 2012): 1.43% (7 participants) in the UO group and 1.64% (8 participants) in the control group; the comprehensive reasons for dropout are provided in Supplementary Table S4. The intervention duration ranged from 14 to 180 days, but no trial reported a follow-up visit. Only one trial applied UO continuously until complete ulcer healing was achieved.

**TABLE 1:** Characteristics of included randomized clinical trials.

Study ID	Types of ulcers	Sample size (U/B)	Age/Year	Sex (M/F)	Comparisons	Treatment duration/d	Outcomes
Ulcer ointment versus no intervention							
Lin et al. (2024)	Venous leg ulcer	80/80	U: 45.29 ± 1.37 B: 45.33 ± 1.40	98/62	Debridement + disinfection + UO Us.ext. qd VS debridement + disinfection + sterile dressing Us.ext. qd	21 d	Ulcer area, MCP-1, HIF-1α, bFGF, VEGF, MVD, LVD
Lin (2015)	Diabetic foot ulcer, pressure ulcer	35/35	U: 54.5 ± 3.0 B: 53 ± 2.5	38/32	Debridement + disinfection + UO Us.ext. qd VS debridement + disinfection + sterile dressing Us.ext. qd	28 d	Healing rate, healing time, clinical effective rate
Xiao (2016)	Acutely infected ulcer	35/35	U: 38.1, 18–65 B: 40.0, 18–65	40/30	Debridement + disinfection + UO Us.ext. qod VS debridement + disinfection + normal saline Us.ext. qod	14 d	Time to normalization of skin temperature, time to pain resolution, ulcer area, efficacy, adverse events
Ulcer ointment versus biomedicine							
Jia (2016)	Diabetic foot ulcer	30/30	U: 70 ± 6.8 B: 69 ± 9.3	33/27	Debridement + disinfection + UO Us.ext. qd VS debridement + disinfection + ELA Us.ext. Qd	14 d	TCM pattern scores, WBC, NE%, ESR, CRP, TGF-β, VEGF, PDGF
Lou (2023)	Diabetic foot ulcer	41/41	30–41: 2; 42–53: 15; 54–95: 22; 66–75: 43	62/20	Debridement + disinfection + UO Us.ext. qod VS debridement + disinfection + rhEGF Us.ext. qod	90 d	Ulcer area, pain scores, Chinese medicine symptom scores, routine blood tests, liver and renal function test
Wang (2008)	Diabetic foot ulcer	14/17	U: 62.7 ± 9.8 B: 62.1 ± 10.5	35/22	Disinfection + UO Us.ext. qd VS disinfection + ELA Us.ext. qd	28 d	Healing rate, efficacy, pain disappearance rate, TcPO2, endothelin, ulcer PH value
Wang et al. (2016)	Venous leg ulcer	50/50	U: 68 ± 25 B: 68 ± 25	53/47	Disinfection + UO Us.ext. qd VS disinfection + ELA Us.ext. qd	60 d	Ulcer area, TNF-α, VEGF, symptom scores, efficacy
Wang et al. (2024)	Diabetic foot ulcer	50/50	U: 62.61 ± 10.87 B: 65.18 ± 10.81	80/20	Debridement + disinfection + UO Us.ext. 2 to 3 times a week VS debridement + disinfection + rhEGF Us.ext. 2 to 3 times a week	84 d	Healing rate, percent change in ulcer area, ulcer area, TCM pattern scores, routine blood tests, liver and renal function test, adverse events
Yang A.H. (2015)	Diabetic foot ulcer	32/28	U: 55.1 ± 12.6 B: 57.9 ± 10.6	38/22	Debridement + disinfection + UO Us.ext. qod VS debridement + disinfection + ELA Us.ext. qod	Until ulcer heals	Healing rate, healing time, clinical effective rate
Yang X.X. (2015)	Diabetic foot ulcer	23/23	U: 63.48 ± 11.26 B: 67.39 ± 9.23	28/18	Disinfection + UO Us.ext. qd VS disinfection + ELA Us.ext. qd	28 d	ABI, ulcer area, pain scores, ulcer scores, TcPO2, WBC, NE%, CRP
Yi (2015)	Venous leg ulcer	30/30	U: 50.00 ± 10.58 B: 55.83 ± 11.43	37/23	Disinfection + UO Us.ext. qd + aescuven forte 300 mg bid po VS disinfection + ELA Us.ext. qd + aescuven forte 300 mg bid po	28 d	Efficacy, ulcer symptom scores (pain, exudation, numbness/pruritus), bacterial culture
Zhang (2013)	Venous leg ulcer	27/24	U: 59.78 ± 15.12 B: 60.05 ± 13.08	24/27	Debridement + disinfection + UO Us.ext. qd/qod VS debridement + disinfection + metronidazole/glucose injection Us.ext. qd/qod	28 d	Efficacy, ulcer area, pain, exudation, numbness, bacterial culture
Zhao (2012)	Diabetic foot ulcer	15/14	40–59: 7 60–78: 22	16/14	Debridement + disinfection + UO Us.ext. qd/qod + antibiotics + microcirculatory agents VS debridement + disinfection + insulin + anisodamine + gentamicin Us.ext. qd/qod + antibiotics + microcirculatory agents	NA	Ulcer area, granulation tissue quality, Wagner grade
Zhu (2014)	Diabetic foot ulcer	27/27	64.7, 49–78	25/29	UO Us.ext. qod + amoxicillin/clavulanate tablets 0.375 g tid po VS petrolatum gauze Us.ext. qod + amoxicillin/clavulanate tablets 0.375 g tid po	180 d	Efficacy

ABI, ankle-brachial index; B, biomedicine; bFGF, basic fibroblast growth factor; CRP, C-reactive protein; ELA, ethacridine lactate; ESR, erythrocyte sedimentation rate; F, female; HIF-1α, hypoxia-inducible factor 1-alpha; LVD, lymphatic vessel density; M, male; MCP-1, monocyte chemoattractant protein-1; MVD, microvessel density; NA, not available; NE%, neutrophil percentage; PDGF, platelet-derived growth factor; qd, once a day; qod, once every other day; rhEGF, recombinant human epidermal growth factor; TCM, traditional Chinese medicine; TcPO2, transcutaneous oxygen pressure; TGF-β, transforming growth factor-beta; TNF-α, tumor necrosis factor-alpha; U/UO, ulcer ointment; VEGF, vascular endothelial growth factor; VS, versus; WBC, white blood cell.

### 3.2 Risk of bias

**FIGURE 2:**
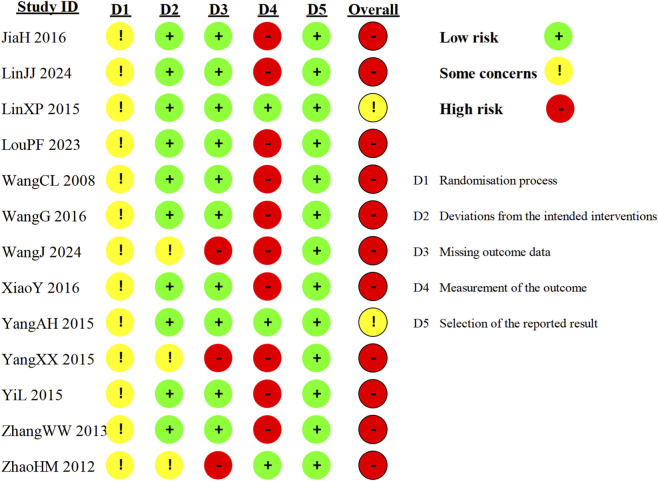
Summary of risk of bias.

For the overall risk of bias, 11 trials were judged as “high” and three were judged as having “some concerns” (Figure 2). All included trials were rated as having “some concerns” in the randomization process domain. Although eight trials reported generating a random sequence using a random number table, none described allocation concealment. For deviations from the intended interventions, most trials were rated “low” risk of bias. No trial utilized blinding of participants and researchers, but since the interventions involving specialized dressings were administered by healthcare workers, it was difficult for participants to deviate from the intended interventions. For missing outcome data, only three trials (Wang et al., 2024; Yang A.H, 2015; Zhao, 2012) had dropouts (ranging from 3.3% to 11%) and were rated “high” risk of bias due to a lack of analysis. The remaining 11 trials were rated “low” risk of bias. For outcome measurement, the four trials (Lin, 2015; Yang A.H, 2015; Zhao, 2012; Zhu, 2014) that assessed objective outcomes were rated “low” risk of bias. The other ten trials, which involved subjective outcomes, were rated “high” risk of bias. For selection of the reported result, all included trials reported their pre-specified outcomes or a relatively complete set of outcomes and were therefore rated “low” risk of bias.

### 3.3 Outcomes of UO versus no intervention

For ulcer healing, UO exhibited a superior effect to no intervention in ulcer healing rate (RR = 2.24, 95% CI: 1.42–3.52, N = 2, n = 140, I^2^ = 0%), ulcer area (MD = –1.85 cm^2^, 95% CI: −2.97 to −0.73, N = 1, n = 70) and healing time (MD = –3.00 days, 95% CI: −4.26 to −1.73, N = 1, n = 70). For symptom alleviation, UO was associated with a greater reduction in pain scores than no intervention (SMD = −0.39, 95% CI: −0.49 to −0.29, N = 1, n = 160). Moreover, serum VEGF levels were significantly higher in the UO group (MD = 22.18 pg/mL, 95% CI: 19.80–24.56, N = 1, n = 160). In contrast, the UO group and the control group had no statistically significant difference in the clinical effective rate (RR = 1.06, 95% CI: 0.98–1.15, N = 2, n = 140, I^2^ = 0%). However, as the findings on healing rate and ulcer area reduction originated from small, single-center trials with methodological weaknesses, the clinical reliability of these findings is limited.

### 3.4 Outcomes of UO versus biomedicine

**FIGURE 3:**
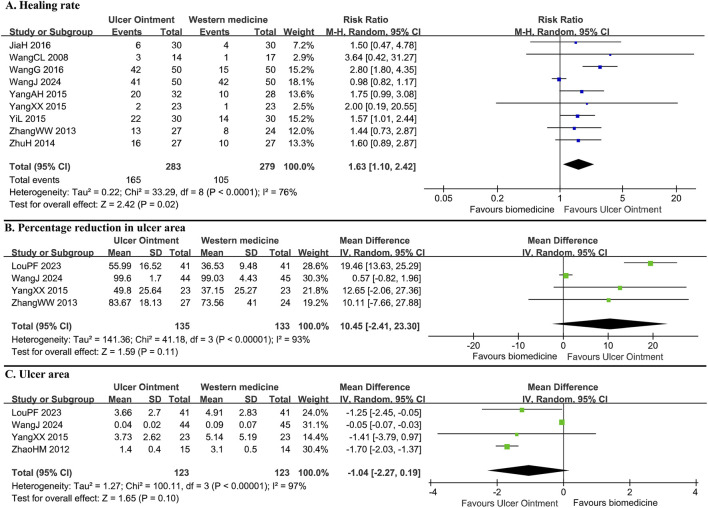
Forest plot for ulcer healing in comparison of Ulcer Ointment versus biomedicine. **(A)** Healing rate; **(B)** Percentage reduction in ulcer area; **(C)** Ulcer area.

For ulcer healing, UO demonstrated a significant advantage over biomedicine in ulcer healing rate (RR = 1.63, 95% CI: 1.10–2.42, N = 9, n = 562, I^2^ = 76%) and healing time (MD = −8.30 days, 95% CI: −9.34 to −7.26, N = 1, n = 60). However, no statistically significant differences were observed between groups in the percentage reduction of ulcer area (MD = 10.45%, 95% CI: −2.41 to 23.30, N = 4, n = 268, I^2^ = 93%) or the absolute ulcer area (MD = −1.04 cm^2^, 95% CI: −2.27 to 0.19, N = 4, n = 246, I^2^ = 97%) (Figure 3). Regarding symptom improvement, UO was superior to biomedicine in reducing pain (SMD = −0.66, 95% CI: −1.10 to −0.22, N = 3, n = 179, I^2^ = 50%) and alleviating TCM symptoms (SMD = −0.85, 95% CI: −1.19 to −0.50, N = 2, n = 142, I^2^ = 0%) (Supplementary Figure S1). The UO group also showed a significantly higher clinical effective rate (RR = 1.18, 95% CI: 1.08–1.28, N = 10, n = 591, I^2^ = 45%) and greater elevation in serum VEGF levels (MD = 23.10 pg/mL, 95% CI: 14.49–31.71, N = 1, n = 100) compared to the biomedicine group.

### 3.5 Subgroup analysis and sensitivity analysis

Subgroup analysis based on ulcer types revealed a statistically significant improvement in the healing rate favoring UO over biomedicine for venous leg ulcers (RR = 1.92, 95% CI: 1.25–2.95, N = 3, n = 211, I^2^ = 54%), whereas no significant benefit was observed for diabetic foot ulcers (RR = 1.41, 95% CI: 0.91–2.19, N = 6, n = 351, I^2^ = 56%). UO demonstrated a higher clinical effective rate than biomedicine in treating both diabetic foot ulcers (RR = 1.21, 95% CI: 1.06–1.38, N = 7, n = 380, I^2^ = 55%) and venous leg ulcers (RR = 1.15, 95% CI: 1.03–1.29, N = 3, n = 211, I^2^ = 31%) (Supplementary Figure S2A).

Sensitivity analysis, which involved excluding a low-quality trial (Wang et al., 2024) with a high dropout rate (11%) and no proper handling of missing data, notably altered the results for the diabetic foot ulcer subgroup. In the comparison of UO versus biomedicine, this exclusion transformed the healing rate from non-significant to statistically significant and reduced heterogeneity to 0% (RR = 1.70, 95% CI: 1.17–2.47, N = 5, n = 251, I^2^ = 0%, Supplementary Figure S3). Similarly, for the same comparison, the results for the percentage reduction in ulcer area (MD = 17.82%, 95% CI: 12.63–23.00, N = 3, n = 179, I^2^ = 0%) and absolute ulcer area (MD = −1.66 cm^2^, 95% CI: −1.98 to −1.35, N = 3, n = 157, I^2^ = 0%) became significant with markedly reduced heterogeneity after the trial’s exclusion. Consequently, this trial was excluded from the final meta-analysis (Table 2).

**TABLE 2:** Summary of effect estimations.

Outcomes	N, n	Estimate effect, 95% CI	*P*
Ulcer ointment versus no intervention			
Healing rate	N = 2, n = 140	RR = 2.24, 1.42 to 3.52, I^2^ = 0%	*P* = 0.0005*
Ulcer area	N = 1, n = 70	MD = −1.85 cm^2^, –2.97 to −0.73	*P* = 0.001*
Healing time	N = 1, n = 70	MD = −3.00 days, −4.26 to −1.73	*P* < 0.00001*
Pain scores	N = 1, n = 160	SMD = −0.39, −0.49 to −0.29	*P* < 0.00001*
Clinical effective rate	N = 2, n = 140	RR = 1.06, 0.98 to 1.15, I^2^ = 0%	*P* = 0.17
VEGF	N = 1, n = 160	MD = 22.18 pg/mL, 19.80 to 24.56	*P* < 0.00001*
Ulcer ointment versus biomedicine			
Healing rate	N = 8, n = 462	RR = 1.87, 1.49 to 2.34, I^2^ = 0%	*P* < 0.00001*
Diabetic foot ulcer	N = 5, n = 251	RR = 1.70, 1.17 to 2.47, I^2^ = 0%	*P* = 0.005*
Venous leg ulcer	N = 3, n = 211	RR = 1.92, 1.25 to 2.95, I^2^ = 54%	*P* = 0.003*
Percentage reduction in ulcer area	N = 3, n = 179	MD = 17.82%, 12.63 to 23.00, I^2^ = 0%	*P* < 0.00001*
Ulcer area	N = 3, n = 157	MD = −1.66 cm^2^, –1.98 to −1.35, I^2^ = 0%	*P* < 0.00001*
Healing time	N = 1, n = 60	MD = −8.30 days, −9.34 to −7.26	*P* < 0.00001*
TCM symptom complex scores	N = 2, n = 142	SMD = −0.85, −1.19 to −0.50, I^2^ = 0%	*P* < 0.00001*
Pain scores	N = 3, n = 179	SMD = −0.66, −1.10 to −0.22, I^2^ = 50%	*P* = 0.003*
Clinical effective rate	N = 9, n = 491	RR = 1.21, 1.10 to 1.32, I^2^ = 40%	*P* < 0.0001*
Diabetic foot ulcer	N = 6, n = 280	RR = 1.26, 1.09 to 1.46, I^2^ = 45%	*P* = 0.002*
Venous leg ulcer	N = 3, n = 211	RR = 1.15, 1.03 to 1.29, I^2^ = 31%	*P* = 0.01*
VEGF	N = 1, n = 100	MD = 23.10 pg/mL, 14.49 to 31.71	*P* < 0.00001*

CI, confidence interval; MD, mean difference; N, number of trials; n, number of patients; *P*, probability value; RR, relative risk; SMD, standard mean difference; VEGF, vascular endothelial growth factor. “*” means that the Ulcer Ointment group is significantly more effective than the biomedicine group.

**FIGURE 4:**
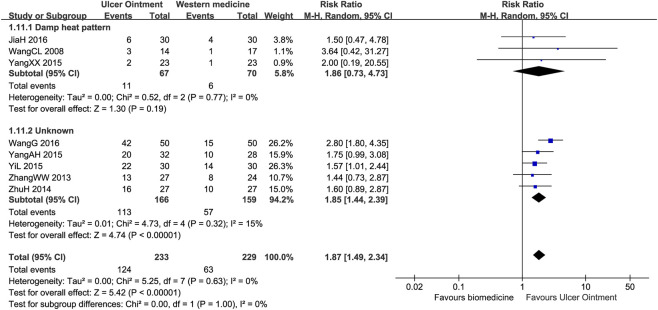
Forest plot of subgroup analysis according to TCM patterns for healing rate in comparison of Ulcer Ointment versus biomedicine.

Subgroup analysis based on TCM patterns indicated that UO was consistently superior to biomedicine in terms of the clinical effective rate, both for patients with a damp-heat pattern (RR = 1.13, 95% CI: 1.01–1.26, N = 3, n = 137, I^2^ = 0%) and those with unknown patterns (RR = 1.23, 95% CI: 1.08–1.39, N = 7, n = 454, I^2^ = 63%) (Supplementary Figure S2B). The effect of UO versus biomedicine on the healing rate, however, varied across TCM patterns. A significant benefit of UO was observed for patients with unknown patterns (RR = 1.85, 95% CI: 1.44–2.39, N = 5, n = 325, I^2^ = 15%), whereas the effect for those with a damp-heat pattern was not statistically significant (RR = 1.86, 95% CI: 0.73–4.73, N = 3, n = 137, I^2^ = 0%), a finding that may be attributed to the limited sample size in the latter subgroup (Figure 4).

### 3.6 Publication bias

**FIGURE 5:**
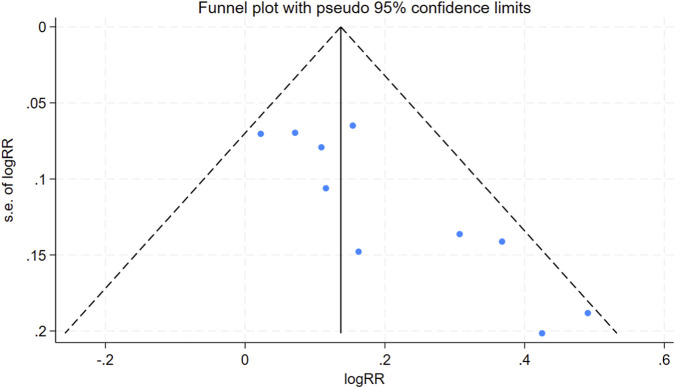
Funnel plot for clinical effective rate (UO versus biomedicine).

For the clinical effective rate in the UO versus biomedicine comparison, the analysis revealed an asymmetrical funnel plot and a statistically significant Egger’s test (N = 10, *P* = 0.003, Figure 5), indicating the presence of significant publication bias. Given the limited sample size, the assessment of publication bias for the healing rate (UO vs. biomedicine) should be considered preliminary. However, the symmetrical funnel plot and non-significant Begg’s test (P = 0.913, Supplementary Figure S4, N = 8) indicated minimal publication bias for the healing rate when comparing UO to biomedicine.

### 3.7 Certainty of evidence

According to the GRADE assessment, the evidence for the superiority of UO over biomedicine or no intervention was rated as low certainty for outcomes including healing rate, percentage reduction in ulcer area, healing time, pain scores, TCM symptom scores, and serum VEGF levels. For ulcer area and clinical effective rate specifically compared to no intervention, the evidence for UO was downgraded to very low certainty due to a high risk of bias, imprecision, and suspected publication bias. A detailed breakdown of the certainty of evidence for each outcome is provided in Supplementary Table S5.

### 3.8 Safety

A total of four trials reported adverse effects. Only one adverse event of mild pruritus (associated with the adhesive tape used for fixation) was reported in each of the UO and the control groups. No severe adverse events or clinically significant abnormalities in laboratory parameters (including liver and kidney function, blood glucose, routine blood tests, and urinalysis) were observed. No local adverse events occurred at the application sites in either group, such as drug allergy, aggravated infection, skin lesions (including rash or blistering), or intolerance. In addition, Wang et al. (2024) reported that the UO group showed significantly higher post-treatment red blood cell count and hemoglobin levels than the rhEGF group (P < 0.001). More details are presented in Supplementary Table S6.

## 4 Discussion

### 4.1 Summary of evidence

This study demonstrated that UO was superior to biomedicine or no intervention for patients with skin ulcers, as it significantly improved healing rate, reduced ulcer area and healing time, alleviated pain, and elevated serum VEGF levels. Furthermore, UO led to a greater percentage reduction in ulcer area, a greater reduction in TCM symptom scores, and a higher clinical effective rate compared to biomedicine. Collectively, the available evidence suggests that UO could be a promising topical intervention for skin ulcers, with initial data indicating an acceptable safety profile. However, given the high risk of bias and low certainty of the included studies, these findings are exploratory and do not yet support routine clinical application.

Subgroup analyses revealed that UO showed no significant difference in improving the healing rate compared to biomedicine for patients with a damp-heat pattern. In contrast, the UO was significantly superior to biomedicine for patients whose ulcers were not characterized by a specific TCM pattern. This discrepancy may be partly attributed to the insufficient sample size in the damp-heat pattern subgroup. An analysis of UO’s botanical drug composition (*Rheum palmatum L.*, *Angelica dahurica*, and *Ligusticum chuanxiong*) based on TCM theory suggests its suitability for treating mixed patterns, such as those involving dampness, heat, blood stasis, and qi deficiency. Its multi-targeted action may be less optimized for a single, specific TCM pattern, which could explain the lack of a significant advantage over biomedicine in the damp-heat pattern subgroup.

### 4.2 Compared with previous studies

Previous systematic reviews have indicated the potential efficacy of various topical TCM agents for specific skin ulcers. For instance, Badu Shengji Powder has been studied for hard-to-heal ulcers (An et al., 2023), Jin Huang Powder for diabetic foot ulcers (Ma et al., 2015), and Resina Draconis for pressure ulcers (Xu et al., 2013). Furthermore, a meta-analysis on botanical drugs for diabetic foot ulcers suggested that topical olive oil and oral bitter melon leaf extract may promote healing, although it did not include any studies on UO (Zamanifard et al., 2024). Nonetheless, these reviews are typically confined to particular ulcer types. In contrast, this study synthesizes evidence from RCTs investigating UO across a diverse spectrum of skin ulcers.

Similar to the results of this review, an RCT demonstrated that the topical application of *Angelica dahurica* was superior to clotrimazole cream in healing pressure ulcers and that its efficacy was associated with significantly elevated levels of VEGF. Furthermore, the same study showed that *Angelica dahurica* upregulates cell viability and clone formation in a dose-dependent manner (Gong et al., 2016).

### 4.3 Strengths and limitations

For strengths, we performed separate meta-analyses for the two distinct comparator types. Regarding outcomes, we focused on healing rate, percent change in ulcer area, ulcer area, and pain score, which represent the most clinically relevant outcomes for skin ulcers. To explore sources of high heterogeneity, we conducted subgroup analyses based on ulcer types and TCM patterns, followed by sensitivity analyses to assess the robustness of the findings. A strength of this review is its broad inclusion criteria, which incorporated RCTs involving a wide spectrum of skin ulcer types—such as diabetic foot ulcers, venous leg ulcers, pressure ulcers, and acute infected ulcers—to comprehensively evaluate the therapeutic potential of UO. However, this inclusive approach also introduced increased clinical heterogeneity into the evidence base, which is an important limitation to consider when interpreting the findings.

For limitations, all included RCTs were conducted in China, and none were placebo-controlled. Thus, external validity to non-Chinese healthcare settings is uncertain. Most of the included trials were assessed as having a “high” risk of bias, exhibiting poor reporting on critical methodological safeguards, particularly regarding allocation concealment and blinding. Besides, substantial heterogeneity existed across the trials, pertaining to variability in UO preparations across hospitals, treatment duration, and outcomes measurements, which further limits the confidence in our pooled results. Due to these methodological shortcomings, the overall certainty of the evidence for the outcomes is low to very low. Consequently, the observed treatment effects are likely to represent an overestimation of the true clinical efficacy. The findings, while indicative of potential benefit, must therefore be regarded as exploratory and hypothesis-generating rather than confirmatory. Furthermore, age variability across included studies is large and the evidence is primarily representative of older populations, and younger adults are markedly underrepresented. We were unable to adequately assess the effect of UO across different phases or in specific TCM patterns.

### 4.4 Clinical and research implications

For future practice, UO may offer therapeutic potential for patients with skin ulcers. According to TCM theory, *Rheum palmatum L.* clears heat and eliminates stasis; *Angelica dahurica* drains pus and regenerates tissues; and *Ligusticum chuanxiong* circulates blood and resolves stagnation. The combination of these three botanical drugs in UO works synergistically to clear heat, promote blood circulation, alleviate pain, and promote tissue regeneration. Furthermore, the oleaginous base of UO, primarily sesame oil which is traditionally used to remove toxins and regenerate new tissues, creates a physical barrier over the ulcer that helps reduce bacterial invasion. Currently, UO is primarily a hospital-prepared formulation that varies across institutions, highlighting the need for standardized manufacturing protocols (Han et al., 2021). In addition, comprehensive therapy, including anti-infection measures, surgical debridement, lower extremity arterial revascularization, negative pressure wound therapy, skin grafting, and prompt multidisciplinary referral, remains essential (Armstrong et al., 2023).

Regarding pharmacological mechanisms, UO significantly promoted healing in rats with diabetic ulcers, potentially through activating the Wnt and Notch signaling pathways (Hao et al., 2022). Similarly, UO alleviated tissue damage in extravasation-induced ulcers by reducing microvascular permeability, vascular inflammation, and edema, mechanisms likely associated with the upregulation of VEGF, epidermal growth factor, and basic fibroblast growth factor (Li et al., 2024). Additionally, key composition of UO, *Angelica dahurica* and *Rheum officinale* extract (ARE) have demonstrated robust efficacy. ARE accelerated ulcer healing in excisional models, exhibiting antimicrobial, anti-inflammatory, and pro-angiogenic activities (Yang et al., 2020). In a high-fat diet–streptozotocin rat model, ARE facilitated ulcer healing and improved glycemic control, which was associated with increased expression of VEGF, α-smooth muscle actin, and inducible nitric oxide synthase, alongside suppressed NF-κB expression (Chao et al., 2021). Besides, *Rheum palmatum L.* possesses anti-inflammatory, antiviral, and antibacterial properties (Yang et al., 2024).

Future research should establish standardized criteria to evaluate the clinical effectiveness of interventions for skin ulcers. Definitive evidence for UO should be generated through multicenter, randomized, double-blind, placebo-controlled trials, supported by pre-registered protocols, transparent reporting, and rigorous monitoring of adverse events, to comprehensively assess the efficacy and safety of UO.

## 5 Conclusion

Based on low-certainty preliminary evidence, this review suggests that UO may serve as a potential topical treatment option for skin ulcers, showing exploratory potential in promoting ulcer healing, alleviating pain, improving TCM symptoms, and elevating serum VEGF levels, with preliminary data also indicating a good safety profile. However, the overall risk of bias in the included studies was high or raised some concerns, and the certainty of the evidence was low or very low for the outcomes. Consequently, these findings remain exploratory and cannot be reliably translated into current clinical practice. The exact efficacy and safety of UO must be confirmed by future high-quality, double-blind RCTs.

## Data Availability

The raw data supporting the conclusions of this article will be made available by the authors, without undue reservation.
