# Supplementary material for: Effectiveness and safety of Chinese traditional medicine Ulcer Ointment for skin ulcers: a systematic review and meta-analysis of randomized controlled trials
Source: Front Pharmacol. 2026 Mar 12;17:1764562. doi: 10.3389/fphar.2026.1764562 (PMC13017936; doi:10.3389/fphar.2026.1764562)
Supplement: Supplementary file 1 [file Supplementaryfile1.docx]

**Supplementary materials**

**
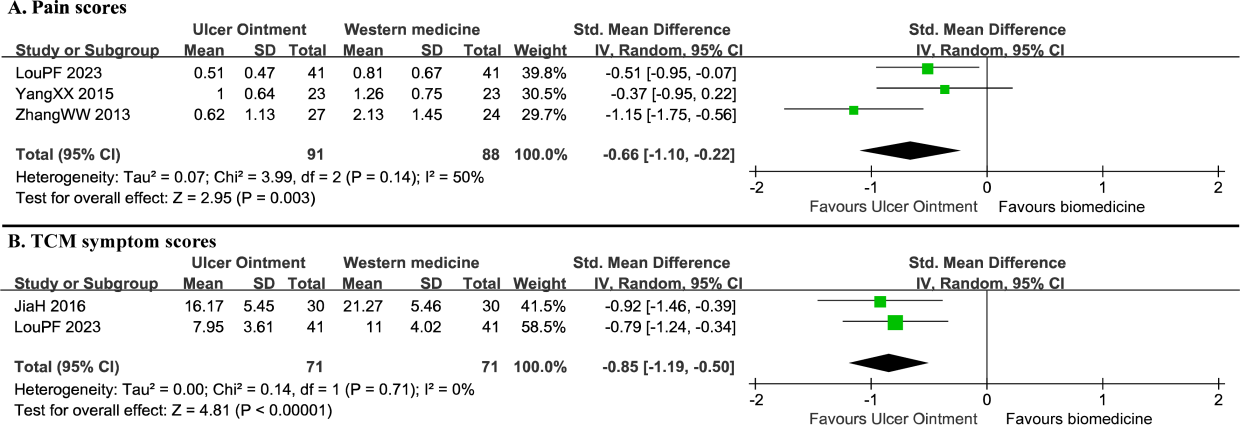
**

**Figure S1.** Forest plot for symptom improvement in comparison of Ulcer Ointment versus biomedicine. (A) Pain scores. (B) TCM symptom scores.

**
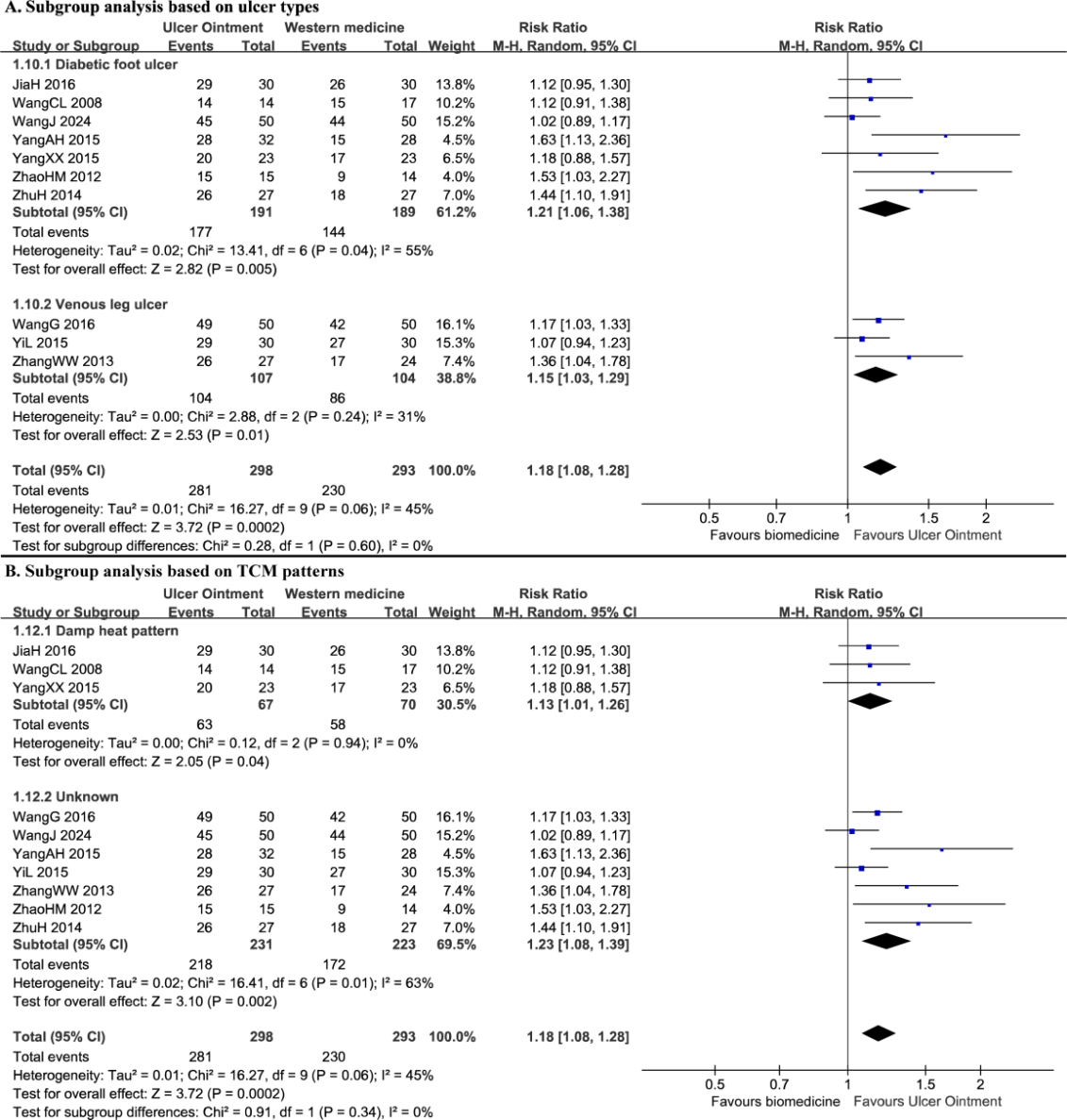
**

**Figure S2** Forest plot of subgroup analysis for clinical effective rate in comparison of Ulcer Ointment versus biomedicine. (A) Subgroup analysis based on ulcer types. (B) Subgroup analysis based on TCM patterns.

**
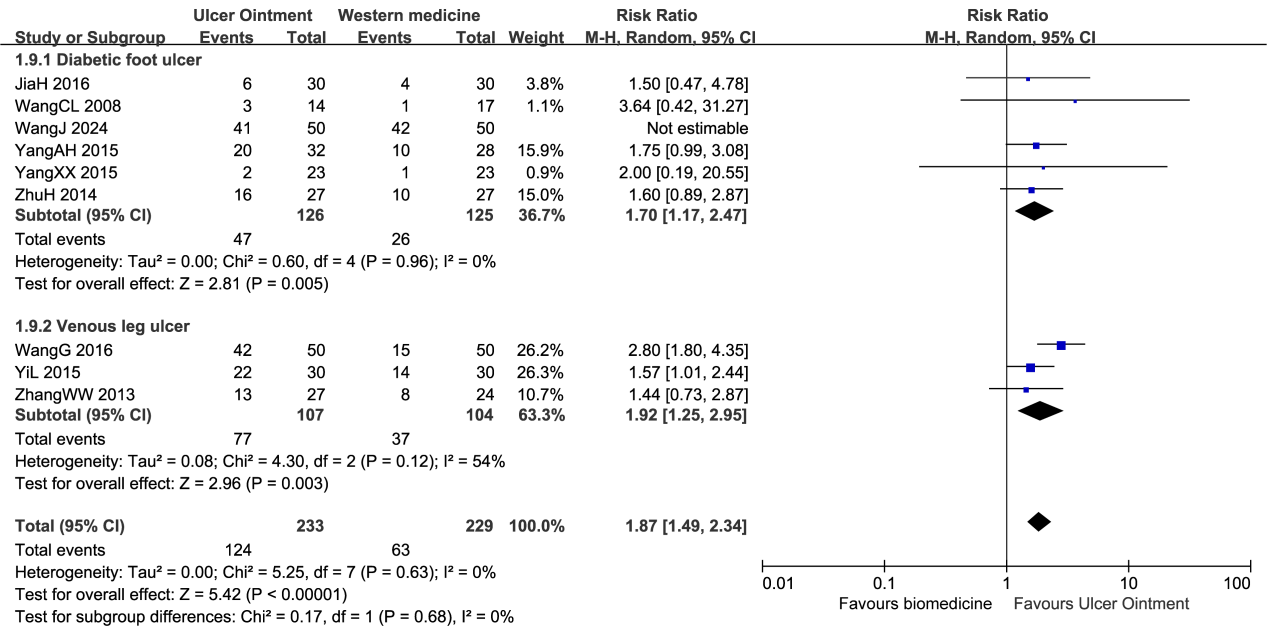
**

**Figure S3** Forest plot and sensitivity analysis of subgroup analysis according to ulcer types for healing rate in comparison of Ulcer Ointment versus biomedicine.

**
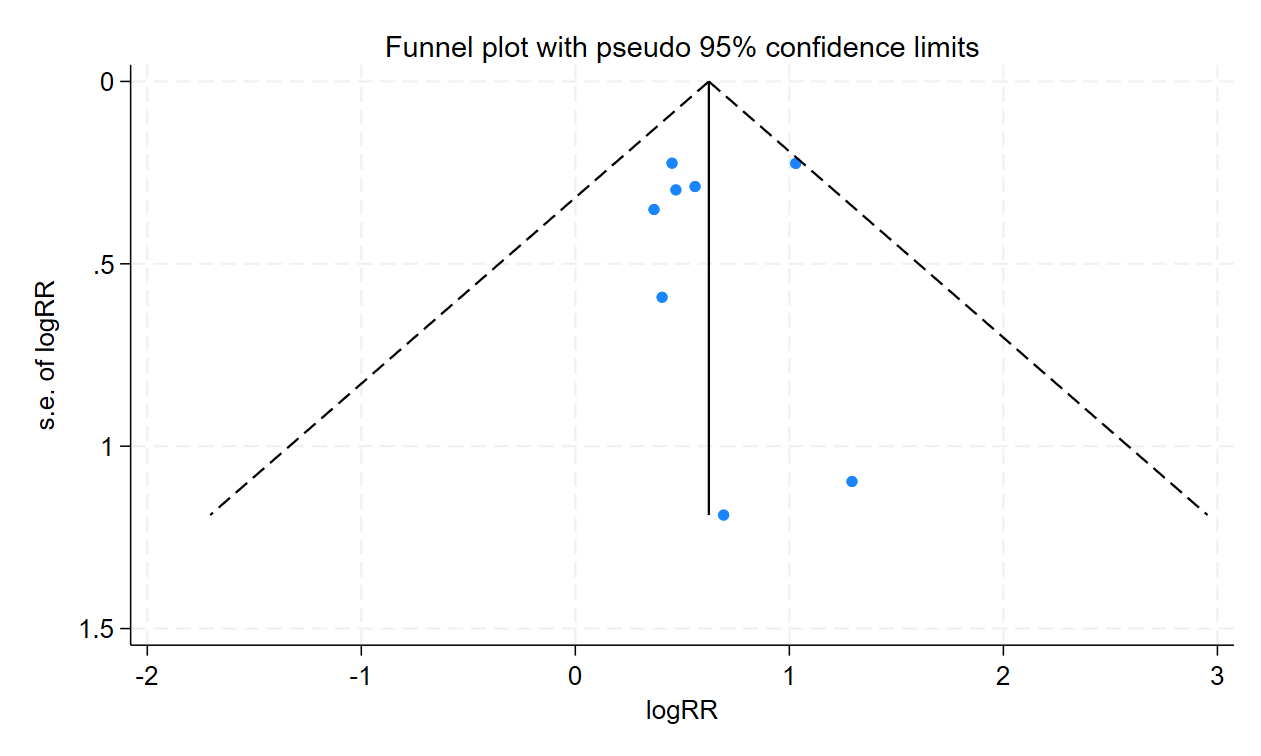
**

**Figure S4.** Funnel plot for healing rate (Ulcer Ointment versus biomedicine).

**Supplementary Table S1** PRISMA 2020 Checklist.

| **Section and Topic** | **Item #** | **Checklist item** | **Location where item is reported** |
| --- | --- | --- | --- |
| **TITLE** | | |  |
| Title | 1 | Identify the report as a systematic review. | Title |
| **ABSTRACT** | | |  |
| Abstract | 2 | See the PRISMA 2020 for Abstracts checklist. | Abstract |
| **INTRODUCTION** | | |  |
| Rationale | 3 | Describe the rationale for the review in the context of existing knowledge. | Introduction |
| Objectives | 4 | Provide an explicit statement of the objective(s) or question(s) the review addresses. | Introduction |
| **METHODS** | | |  |
| Eligibility criteria | 5 | Specify the inclusion and exclusion criteria for the review and how studies were grouped for the syntheses. | 2.2 |
| Information sources | 6 | Specify all databases, registers, websites, organisations, reference lists and other sources searched or consulted to identify studies. Specify the date when each source was last searched or consulted. | 2.3 |
| Search strategy | 7 | Present the full search strategies for all databases, registers and websites, including any filters and limits used. | Table S2 |
| Selection process | 8 | Specify the methods used to decide whether a study met the inclusion criteria of the review, including how many reviewers screened each record and each report retrieved, whether they worked independently, and if applicable, details of automation tools used in the process. | 2.4 |
| Data collection process | 9 | Specify the methods used to collect data from reports, including how many reviewers collected data from each report, whether they worked independently, any processes for obtaining or confirming data from study investigators, and if applicable, details of automation tools used in the process. | 2.4 |
| Data items | 10a | List and define all outcomes for which data were sought. Specify whether all results that were compatible with each outcome domain in each study were sought (e.g. for all measures, time points, analyses), and if not, the methods used to decide which results to collect. | 2.2.4 |
|  | 10b | List and define all other variables for which data were sought (e.g. participant and intervention characteristics, funding sources). Describe any assumptions made about any missing or unclear information. | 2.4 |
| Study risk of bias assessment | 11 | Specify the methods used to assess risk of bias in the included studies, including details of the tool(s) used, how many reviewers assessed each study and whether they worked independently, and if applicable, details of automation tools used in the process. | 2.5 |
| Effect measures | 12 | Specify for each outcome the effect measure(s) (e.g. risk ratio, mean difference) used in the synthesis or presentation of results. | 2.6 |
| Synthesis methods | 13a | Describe the processes used to decide which studies were eligible for each synthesis (e.g. tabulating the study intervention characteristics and comparing against the planned groups for each synthesis (item #5)). | 2.6 |
|  | 13b | Describe any methods required to prepare the data for presentation or synthesis, such as handling of missing summary statistics, or data conversions. | NA |
|  | 13c | Describe any methods used to tabulate or visually display results of individual studies and syntheses. | 2.6 |
|  | 13d | Describe any methods used to synthesize results and provide a rationale for the choice(s). If meta-analysis was performed, describe the model(s), method(s) to identify the presence and extent of statistical heterogeneity, and software package(s) used. | 2.6 |
|  | 13e | Describe any methods used to explore possible causes of heterogeneity among study results (e.g. subgroup analysis, meta-regression). | 2.6 |
|  | 13f | Describe any sensitivity analyses conducted to assess robustness of the synthesized results. | 2.6 |
| Reporting bias assessment | 14 | Describe any methods used to assess risk of bias due to missing results in a synthesis (arising from reporting biases). | 2.6 |
| Certainty assessment | 15 | Describe any methods used to assess certainty (or confidence) in the body of evidence for an outcome. | 2.6 |
| **RESULTS** | | |  |
| Study selection | 16a | Describe the results of the search and selection process, from the number of records identified in the search to the number of studies included in the review, ideally using a flow diagram. | Figure 1 |
|  | 16b | Cite studies that might appear to meet the inclusion criteria, but which were excluded, and explain why they were excluded. | Figure 1 |
| Study characteristics | 17 | Cite each included study and present its characteristics. | Table 1 |
| Risk of bias in studies | 18 | Present assessments of risk of bias for each included study. | Figure 2 |
| Results of individual studies | 19 | For all outcomes, present, for each study: (a) summary statistics for each group (where appropriate) and (b) an effect estimate and its precision (e.g. confidence/credible interval), ideally using structured tables or plots. | Table 2 |
| Results of syntheses | 20a | For each synthesis, briefly summarise the characteristics and risk of bias among contributing studies. | 3.2, Table S5 |
|  | 20b | Present results of all statistical syntheses conducted. If meta-analysis was done, present for each the summary estimate and its precision (e.g. confidence/credible interval) and measures of statistical heterogeneity. If comparing groups, describe the direction of the effect. | 3.3, 3.4,  Table 2 |
|  | 20c | Present results of all investigations of possible causes of heterogeneity among study results. | 3.5 |
|  | 20d | Present results of all sensitivity analyses conducted to assess the robustness of the synthesized results. | 3.5 |
| Reporting biases | 21 | Present assessments of risk of bias due to missing results (arising from reporting biases) for each synthesis assessed. | 3.6 |
| Certainty of evidence | 22 | Present assessments of certainty (or confidence) in the body of evidence for each outcome assessed. | 3.7, Table S5 |
| **DISCUSSION** | | |  |
| Discussion | 23a | Provide a general interpretation of the results in the context of other evidence. | 4.1 |
|  | 23b | Discuss any limitations of the evidence included in the review. | 4.3 |
|  | 23c | Discuss any limitations of the review processes used. | 4.3 |
|  | 23d | Discuss implications of the results for practice, policy, and future research. | 4.4 |
| **OTHER INFORMATION** | | |  |
| Registration and protocol | 24a | Provide registration information for the review, including register name and registration number, or state that the review was not registered. | 2.1 |
|  | 24b | Indicate where the review protocol can be accessed, or state that a protocol was not prepared. | 2.1 |
|  | 24c | Describe and explain any amendments to information provided at registration or in the protocol. | NA |
| Support | 25 | Describe sources of financial or non-financial support for the review, and the role of the funders or sponsors in the review. | Funding |
| Competing interests | 26 | Declare any competing interests of review authors. | Conflict of Interest |
| Availability of data, code and other materials | 27 | Report which of the following are publicly available and where they can be found: template data collection forms; data extracted from included studies; data used for all analyses; analytic code; any other materials used in the review. | Data availability |

**Supplementary Table S2** Searching strategies.

| **Database searched** | **Records** |
| --- | --- |
| Chinese National Knowledge Infrastructure Database (CNKI) | 57 |
| Wanfang Database | 12 |
| Chinese Science and Technique Journals Database (VIP) | 17 |
| Chinese Biomedical Literature Database (Sinomed) | 7 |
| Yiigle databases | 0 |
| PubMed | 0 |
| Embase | 0 |
| Cochrane library | 0 |
| **Total** | **93** |

**Searching time: October 28, 2025**

**CNKI**

TKA=('随机' + '随机对照') AND TKA=('糖尿病足溃疡' + '血管性溃疡' + '下肢静脉性溃疡' + '下肢动脉闭塞硬化性溃疡' + '创伤性溃疡' + '压力性溃疡' + '慢性溃疡' + '皮肤溃疡') AND (TKA='溃疡油' OR (FT='大黄' AND FT='白芷' AND FT='川芎'))

**Wanfang Database**

(题名或关键词:(随机 OR 随机对照) or 摘要:(随机 OR 随机对照)) AND (题名或关键词:(糖尿病足溃疡 OR 血管性溃疡 OR 下肢静脉性溃疡 OR 下肢动脉闭塞硬化性溃疡 OR 创伤性溃疡 OR 压力性溃疡 OR 慢性溃疡 OR 皮肤溃疡) or 摘要:(糖尿病足溃疡 OR 血管性溃疡 OR 下肢静脉性溃疡 OR 下肢动脉闭塞硬化性溃疡 OR 创伤性溃疡 OR 压力性溃疡 OR 慢性溃疡 OR 皮肤溃疡)) AND ((摘要:("溃疡油") or 题名或关键词:("溃疡油")) OR 全部:(大黄 AND 白芷 AND 川芎))

**VIP**

篇关摘=随机 OR 随机对照 AND 篇关摘=糖尿病足溃疡 R 血管性溃疡 OR 下肢静脉性溃疡 OR 下肢动脉闭塞硬化性溃疡 OR 创伤性溃疡 OR 压力性溃疡 OR 慢性溃疡 OR 皮肤溃疡 AND 篇关摘=溃疡油

**Sinomed**

 (( "糖尿病足溃疡"[摘要:智能] OR "血管性溃疡"[摘要:智能] OR "下肢静脉性溃疡"[摘要:智能] OR "下肢动脉闭塞硬化性溃疡"[摘要:智能] OR "创伤性溃疡"[摘要:智能] OR "压力性溃疡"[摘要:智能] OR "慢性溃疡"[摘要:智能] OR "皮肤溃疡"[摘要:智能]) AND( "随机"[摘要:智能] OR "随机对照"[摘要:智能]) AND "溃疡油"[摘要:智能]) OR (( "糖尿病足溃疡"[摘要:智能] OR "血管性溃疡"[摘要:智能] OR "下肢静脉性溃疡"[摘要:智能] OR "下肢动脉闭塞硬化性溃疡"[摘要:智能] OR "创伤性溃疡"[摘要:智能] OR "压力性溃疡"[摘要:智能] OR "慢性溃疡"[摘要:智能] OR "皮肤溃疡"[摘要:智能]) AND( "随机"[摘要:智能] OR "随机对照"[摘要:智能]) AND( "大黄"[摘要:智能] AND "白芷"[摘要:智能] AND "川芎"[摘要:智能]))

**Yiigle databases**

((M=糖尿病足溃疡 OR 血管性溃疡 OR 下肢静脉性溃疡 OR 下肢动脉闭塞硬化性溃疡 OR 创伤性溃疡 OR 压力性溃疡 OR 慢性溃疡 OR 皮肤溃疡) AND (M=溃疡油) AND (M=随机 OR 随机对照)) OR ((M=糖尿病足溃疡 OR 血管性溃疡 OR 下肢静脉性溃疡 OR 下肢动脉闭塞硬化性溃疡 OR 创伤性溃疡 OR 压力性溃疡 OR 慢性溃疡 OR 皮肤溃疡) AND (ALL=大黄 AND 白芷 AND 川芎) AND (M=随机 OR 随机对照))

**Pubmed**

((Ulcer*[Title/Abstract]) AND ((RCT[Title/Abstract]) OR (random*[Title/Abstract]))) AND ((((((((Rheum) OR (officinale Baill)) OR (tanguticum Maxim)) OR (rhubarb root)) OR (Dahuang)) OR (Da Huang)) AND (((Angelica dahurica) OR (Baizhi)) OR (Bai Zhi))) AND (((Chuanxiong) OR (Szechuan Lovage)) OR (Chuan Xiong)))

**Embase**

((Rheum OR 'officinale Baill' OR 'tanguticum Maxim' OR 'rhubarb root' OR Dahuang OR 'Da Huang') AND ('Angelica dahurica' OR 'Bai Zhi' OR Baizhi OR) AND (Chuanxiong OR 'Szechuan Lovage' OR 'Chuan Xiong')) AND Ulcer*:ti,ab,kw AND (randomized:ti,ab,kw OR rct:ti,ab,kw)

**Cochrane library**

#1 Ulcer*

#2 random*

#3 RCT

#4 #2 OR #3

#5 Rheum

#6 officinale Baill

#7 tanguticum Maxim

#8 rhubarb root

#9 Dahuang

#10 Da Huang

#11 #5 OR #6 OR #7 OR #8 OR #9 OR #10

#12 Angelica dahurica

#13 Baizhi

#14 Bai Zhi

#15 #12 OR #13 OR #14

#16 Chuanxiong

#17 Szechuan Lovage

#18 Chuan Xiong

#19 #16 OR #17 OR #18

#20 #11 AND #15 AND #19

#21 #4 AND #1 AND #20

**Supplementary Table S3** Preparation process of Ulcer Ointment in included studies.

| **Study ID** | **Preparation process** | **Approval numbers** | **Manufacturers** |
| --- | --- | --- | --- |
| JiaH 2016 | *Rheum palmatum* L. [Polygonaceae; *Rhei radix et rhizoma*] 80g, *Angelica dahurica* (Fisch. ex Hoffm.) Benth. & Hook.f. ex Franch. & Sav. [Apiaceae; *Angelicae dahuricae radix*] 80g, *Ligusticum chuanxiong* Hort. [Apiaceae; *Chuanxiong rhizoma*] 80g. The above three botanical drugs are appropriately crushed, take 1200g of sesame oil, combine them in a pot, fry until the botanical drugs dried, discard the dregs, filter, package, sterilize. | Z20053157, Beijing | Dongzhimen Hospital, Beijing University of Chinese Medicine, Beijing, China |
| LinJJ 2024 | *Rheum palmatum* L. [Polygonaceae; *Rhei radix et rhizoma*] 80g, *Angelica dahurica* (Fisch. ex Hoffm.) Benth. & Hook.f. ex Franch. & Sav. [Apiaceae; *Angelicae dahuricae radix*] 80g, *Ligusticum chuanxiong* Hort. [Apiaceae; *Chuanxiong rhizoma*] 80g. The above three botanical drugs are appropriately crushed, take 1200g of sesame oil, combine them in a pot, fry until the botanical drugs dried, discard the dregs, filter, package, sterilize. | 20170001,Longquan | Longquan People's Hospital, Zhejiang, China |
| LinXP 2015 | 80 g each of *Rheum palmatum* L. [Polygonaceae; *Rhei radix et rhizoma*], *Angelica dahurica* (Fisch. ex Hoffm.) Benth. & Hook.f. ex Franch. & Sav. [Apiaceae; *Angelicae dahuricae radix*], *Ligusticum chuanxiong* Hort. [Apiaceae; *Chuanxiong rhizoma*] were decocted in 1000 mL sesame oil for 1 h. After removing impurities, sterile gauze was immersed in the filtrate for use. | NA | Tiantai County Hospital of Traditional Chinese Medicine, Zhejiang, China |
| LouPF 2023 | *Rheum palmatum* L. [Polygonaceae; *Rhei radix et rhizoma*] 80g, *Angelica dahurica* (Fisch. ex Hoffm.) Benth. & Hook.f. ex Franch. & Sav. [Apiaceae; *Angelicae dahuricae radix*] 80g, *Ligusticum chuanxiong* Hort. [Apiaceae; *Chuanxiong rhizoma*] 80g. The above three botanical drugs are appropriately crushed, take 1200g of sesame oil, combine them in a pot, fry until the botanical drugs dried, discard the dregs, filter, package, sterilize. | Z20053817, Beijing | China-Japan Friendship Hospital, Beijing, China |
| WangCL 2008 | *Rheum palmatum* L. [Polygonaceae; *Rhei radix et rhizoma*] 80g, *Angelica dahurica* (Fisch. ex Hoffm.) Benth. & Hook.f. ex Franch. & Sav. [Apiaceae; *Angelicae dahuricae radix*] 80g, *Ligusticum chuanxiong* Hort. [Apiaceae; *Chuanxiong rhizoma*] 80g. The above three botanical drugs are appropriately crushed, take 1200g of sesame oil, combine them in a pot, fry until the botanical drugs dried, discard the dregs, filter, package, sterilize. | F-1259 052, Beijing | Dongzhimen Hospital, Beijing University of Chinese Medicine, Beijing, China |
| WangG 2016 | *Rheum palmatum* L. [Polygonaceae; *Rhei radix et rhizoma*] 80g, *Angelica dahurica* (Fisch. ex Hoffm.) Benth. & Hook.f. ex Franch. & Sav. [Apiaceae; *Angelicae dahuricae radix*] 80g, and *Ligusticum chuanxiong* Hort. [Apiaceae; *Chuanxiong rhizoma*] 80g were soaked in 1200 mL sesame oil for 24 h, followed by low-heat decoction for 1 h. The cooled mixture was then filtered. | Z20053157, Beijing | Dongzhimen Hospital, Beijing University of Chinese Medicine, Beijing, China |
| WangJ 2024 | *Rheum palmatum* L. [Polygonaceae; *Rhei radix et rhizoma*] 80g, *Angelica dahurica* (Fisch. ex Hoffm.) Benth. & Hook.f. ex Franch. & Sav. [Apiaceae; *Angelicae dahuricae radix*] 80g, *Ligusticum chuanxiong* Hort. [Apiaceae; *Chuanxiong rhizoma*] 80g. The above three botanical drugs are appropriately crushed, take 1200g of sesame oil, combine them in a pot, fry until the botanical drugs dried, discard the dregs, filter, package, sterilize. | Z20053817, Beijing | China-Japan Friendship Hospital, Beijing, China |
| XiaoY 2016 | *Rheum palmatum* L. [Polygonaceae; *Rhei radix et rhizoma*] 80g, *Angelica dahurica* (Fisch. ex Hoffm.) Benth. & Hook.f. ex Franch. & Sav. [Apiaceae; *Angelicae dahuricae radix*] 80g, *Ligusticum chuanxiong* Hort. [Apiaceae; *Chuanxiong rhizoma*] 80g. The above three botanical drugs are appropriately crushed, take 1200g of sesame oil, combine them in a pot, fry until the botanical drugs dried, discard the dregs, filter, package, sterilize. | NA | Dongzhimen Hospital, Beijing University of Chinese Medicine, Beijing, China |
| YangAH 2015 | *Rheum palmatum* L. [Polygonaceae; *Rhei radix et rhizoma*] 80g, *Angelica dahurica* (Fisch. ex Hoffm.) Benth. & Hook.f. ex Franch. & Sav. [Apiaceae; *Angelicae dahuricae radix*] 80g, *Ligusticum chuanxiong* Hort. [Apiaceae; *Chuanxiong rhizoma*] 80g. The above three botanical drugs are appropriately crushed, take 1200g of sesame oil, combine them in a pot, fry until the botanical drugs dried, discard the dregs, filter, package, sterilize. | NA | Dongzhimen Hospital, Beijing University of Chinese Medicine, Beijing, China |
| YangXX 2015 | *Rheum palmatum* L. [Polygonaceae; *Rhei radix et rhizoma*] 80g, *Angelica dahurica* (Fisch. ex Hoffm.) Benth. & Hook.f. ex Franch. & Sav. [Apiaceae; *Angelicae dahuricae radix*] 80g, *Ligusticum chuanxiong* Hort. [Apiaceae; *Chuanxiong rhizoma*] 80g. The above three botanical drugs are appropriately crushed, take 1200g of sesame oil, combine them in a pot, fry until the botanical drugs dried, discard the dregs, filter, package, sterilize. | Z20053817, Beijing | Dongzhimen Hospital, Beijing University of Chinese Medicine, Beijing, China |
| YiL 2015 | *Rheum palmatum* L. [Polygonaceae; *Rhei radix et rhizoma*] 80g, *Angelica dahurica* (Fisch. ex Hoffm.) Benth. & Hook.f. ex Franch. & Sav. [Apiaceae; *Angelicae dahuricae radix*] 80g, *Ligusticum chuanxiong* Hort. [Apiaceae; *Chuanxiong rhizoma*] 80g. The above three botanical drugs are appropriately crushed, take 1200g of sesame oil, combine them in a pot, fry until the botanical drugs dried, discard the dregs, filter, package, sterilize. | 120017,  Beijing | Dongzhimen Hospital, Beijing University of Chinese Medicine, Beijing, China |
| ZhangWW 2013 | *Rheum palmatum* L. [Polygonaceae; *Rhei radix et rhizoma*] 80g, *Angelica dahurica* (Fisch. ex Hoffm.) Benth. & Hook.f. ex Franch. & Sav. [Apiaceae; *Angelicae dahuricae radix*] 80g, *Ligusticum chuanxiong* Hort. [Apiaceae; *Chuanxiong rhizoma*] 80g. The above three botanical drugs are appropriately crushed, take 1200g of sesame oil and combine, fry until the botanical drugs dried, discard the dregs, filter, package, sterilize, and the UO is ready. | 120017,  Beijing | Dongzhimen Hospital, Beijing University of Chinese Medicine, Beijing, China |
| ZhaoHM 2012 | *Rheum palmatum* L. [Polygonaceae; *Rhei radix et rhizoma*], *Angelica dahurica* (Fisch. ex Hoffm.) Benth. & Hook.f. ex Franch. & Sav. [Apiaceae; *Angelicae dahuricae radix*], and *Ligusticum chuanxiong* Hort. [Apiaceae; *Chuanxiong rhizoma*] were soaked and then decocted in sesame oil. The dregs were removed, and the oil was sterilized with sterile gauze pads. | NA | Zhecheng County Hospital of Traditional Chinese Medicine, Henan, China |
| ZhuH 2014 | 280 g each of *Rheum palmatum* L. [Polygonaceae; *Rhei radix et rhizoma*], *Angelica dahurica* (Fisch. ex Hoffm.) Benth. & Hook.f. ex Franch. & Sav. [Apiaceae; *Angelicae dahuricae radix*], and *Ligusticum chuanxiong* Hort. [Apiaceae; *Chuanxiong rhizoma*] were soaked in 3000 mL of bean-peanut oil for 3 days, boiled for 30 min, and filtered to obtain Ulcer Ointment. | NA | Qinghe Community Health Service Center, Haidian District, Beijing, China |

**Abbreviations:** NA, Not Available.

**Supplementary Table S4** Dropouts and reasons.

| StudyID | n | Dropouts (%) | Reasons for dropouts |
| --- | --- | --- | --- |
| WangJ 2024 | 50/50 | 11 (11) | U: 6 loss to follow-up; B: 5 loss to follow-up |
| YangXX 2015 | 25/25 | 4 (8) | U: 1 for geographical distance; 1 for perceiving lack of efficacy;  B: 1 for amputation due to critical ischemia, 1 for lack of efficacy. |
| ZhaoHM 2012 | 15/15 | 1 (3.3) | B: 1 for early discharge because of financial problems. |

**Abbreviations:** B, biomedicine; n, number of patients; U, Ulcer Ointment group.

**Supplementary Table S5** GRADE assessment of the effects of Ulcer Ointment on skin ulcers in this meta-analysis.

| **Outcomes** | **n (N)** | **Effect Estimates (95%CI)** | **Risk of Bias** | **Inconsistency** | **Indirectness** | **Imprecision** | **Publication Bias** | **Quality** |
| --- | --- | --- | --- | --- | --- | --- | --- | --- |
| **Ulcer Ointment versus No intervention** | | | | | | | | |
| Healing rate | 140 (2) | RR 2.24 (1.42 to 3.52) | Very Serious^2^ | Not Serious | Not Serious | Not Serious | Undetected | **LOW** |
| Ulcer area | 70 (1) | MD -1.85 cm^2^ (-2.97 to -0.73) | Very Serious^2^ | Not Serious | Not Serious | Serious^3^ | Undetected | **VERY LOW** |
| Healing time | 70 (1) | MD -3.00 days (-4.26 to -1.73) | Serious^1^ | Not Serious | Not Serious | Serious^3^ | Undetected | **LOW** |
| Pain score | 160 (1) | SMD -0.39 (-0.49 to -0.29) | Very Serious^2^ | Not Serious | Not Serious | Not Serious | Undetected | **LOW** |
| Clinical effective rate | 140 (2) | RR 1.06 (0.98 to 1.15) | Very Serious^2^ | Not Serious | Not Serious | Serious^4^ | Undetected | **VERY LOW** |
| VEGF | 160 (1) | MD 22.18 pg/ml (19.80 to 24.56) | Very Serious^2^ | Not Serious | Not Serious | Not Serious | Undetected | **LOW** |
| **Ulcer Ointment versus biomedicine** | | | | | | | | |
| Healing rate | 462 (8) | RR 1.87 (1.49 to 2.34) | Very Serious^2^ | Not Serious | Not Serious | Not Serious | Undetected | **LOW** |
| Percent change in ulcer area | 179 (3) | MD 17.82 % (12.63 to 23.00) | Very Serious^2^ | Not Serious | Not Serious | Not Serious | Undetected | **LOW** |
| Ulcer area | 157 (3) | MD -1.66 cm^2^ (-1.98 to -1.35) | Very Serious^2^ | Not Serious | Not Serious | Not Serious | Undetected | **LOW** |
| Healing time | 60 (1) | MD -8.3 days (-9.34 to -7.26) | Serious^1^ | Not Serious | Not Serious | Serious^3^ | Undetected | **LOW** |
| TCM symptom score | 142 (2) | SMD -0.85 (-1.19 to -0.50) | Very Serious^2^ | Not Serious | Not Serious | Not Serious | Undetected | **LOW** |
| Pain score | 179 (3) | SMD -0.66 (-1.10 to -0.22) | Very Serious^2^ | Not Serious | Not Serious | Not Serious | Undetected | **LOW** |
| Clinical effective rate | 491 (9) | RR 1.21 (1.10 to 1.32) | Very Serious^2^ | Not Serious | Not Serious | Not Serious | Strongly Suspected^5^ | **VERY LOW** |
| VEGF | 100 (1) | MD 23.10 pg/ml (14.49 to 31.71) | Very Serious^2^ | Not Serious | Not Serious | Not Serious | Undetected | **LOW** |

**Abbreviations:** CI, confidence interval; MD, mean difference; N, number of trials; n, number of patients; RR, relative risk; SMD, standard mean difference; TCM, traditional Chinese medicine; VEGF, vascular endothelial growth factor; 1, Some concern risk of bias in the majority of trials; 2, High risk of bias in the majority of trials; 3, Limited sample size; 4, No significant difference between groups; 5, Egger's test was significant in publication bias (p<0.05).

**Supplementary Table S6** Adverse events of Ulcer Ointment for skin ulcer.

| **Study ID** | **Types of ulcers** | **Sample size test / control** | **Incidence** | **Outcomes** |
| --- | --- | --- | --- | --- |
| LouPF 2023 | Diabetic foot ulcer | 41/41 | 0 | No significant abnormalities in liver/kidney function or routine blood tests.  No drug allergy. |
| WangCL 2008 | Diabetic foot ulcer | 14/17 | 0 | No significant abnormalities in liver and kidney function, blood glucose, routine blood tests, or urinalysis. |
| WangJ 2024 | Diabetic foot ulcer | 50/50 | 0 | Post-treatment RBC count and hemoglobin levels were significantly higher in the UO group than in the control group (P＜0.001).  No significant abnormalities in liver or kidney function.  No cases of drug allergy, aggravated infection, or skin damage. |
| XiaoY 2016 | Acutely infected ulcer | 35/35 | U: 1  B: 1 | Incidence of mild tape-site pruritus: 1 case in each group.  No local skin lesions (rash or blistering) or intolerance occurred at the drug application sites in either group, and no toxic or side effects were observed. |

**Abbreviations:** B, biomedicine; RBC, Red blood cell; U, Ulcer Ointment.
